# Epidemiology, Seasonality and Factors Associated with Rotavirus Infection among Children with Moderate-to-Severe Diarrhea in Rural Western Kenya, 2008–2012: The Global Enteric Multicenter Study (GEMS)

**DOI:** 10.1371/journal.pone.0160060

**Published:** 2016-08-05

**Authors:** Richard Omore, Jacqueline E. Tate, Ciara E. O’Reilly, Tracy Ayers, John Williamson, Feny Moke, Katie A. Schilling, Alex O. Awuor, Peter Jaron, John B. Ochieng, Joseph Oundo, Umesh D. Parashar, Michele B. Parsons, Cheryl C. Bopp, Dilruba Nasrin, Tamer H. Farag, Karen L. Kotloff, James P. Nataro, Sandra Panchalingam, Myron M. Levine, Kayla F. Laserson, J. Pekka Nuorti, Eric D. Mintz, Robert F. Breiman

**Affiliations:** 1 Centre for Global Health Research, Kenya Medical Research Institute, Kisumu, Kenya; 2 Division of Viral Diseases, US Centers for Disease Control and Prevention, Atlanta, GA, United States of America; 3 Division of Foodborne, Waterborne and Environmental Diseases, US Centers for Disease Control and Prevention, Atlanta, GA, United States of America; 4 US Centers for Disease Control and Prevention, Kisumu, Kenya; 5 Global Disease Detection Division, Kenya Office of the US Centers for Disease Control and Prevention, Nairobi, Kenya; 6 University of Maryland, School of Medicine, Center for Vaccine Development, Baltimore, MD, United States of America; 7 Department of Pediatrics, University of Virginia School of Medicine, Charlottesville, VA, United States of America; 8 CDC India, Delhi, India; 9 University of Tampere School of Health Sciences, Tampere, Finland; 10 Emory Global Health Institute, Emory University, Atlanta Georgia, United States of America; New York City Department of Health and Mental Hygiene, UNITED STATES

## Abstract

**Objective:**

To evaluate factors associated with rotavirus diarrhea and to describe severity of illness among children <5 years old with non-dysenteric, moderate-to-severe diarrhea (MSD) in rural western Kenya.

**Methods:**

We analyzed data from children <5 years old with non-dysenteric MSD enrolled as cases in the Global Enteric Multicenter Study (GEMS) in Kenya. A non-dysenteric MSD case was defined as a child with ≥3 loose stools in 24 hrs. and one or more of the following: sunken eyes, skin tenting, intravenous rehydration, or hospitalization, who sought care at a sentinel health center within 7 days of illness onset. Rotavirus antigens in stool samples were detected by ELISA. Demographic and clinical information was collected at enrollment and during a single follow-up home visit at approximately 60 days. We analyzed diarrhea severity using a GEMS 17 point numerical scoring system adapted from the Vesikari score. We used logistic regression to evaluate factors associated with rotavirus infection.

**Results:**

From January 31, 2008 to September 30, 2012, among 1,637 (92%) non-dysenteric MSD cases, rotavirus was detected in stools of 245 (15.0%). Rotavirus-positive compared with negative cases were: younger (median age, 8 vs. 13 months; *p*<0.0001), had more severe illness (median severity score, 9 vs 8; *p*<0.0001) and had to be hospitalized more frequently (37/245 [15.1%] vs. 134/1,392 [9.6%]), *p* <0.013). Independent factors associated with rotavirus infection included age 0–11 months old (aOR = 5.29, 95% CI 3.14–8.89) and presenting with vomiting ≥3 times/24hrs (aOR = 2.58, 95% CI [1.91–3.48]). Rotavirus was detected more commonly in warm and dry months than in the cool and rainy months (142/691 [20%] vs 70/673 [10%]) *p*<0.0001).

**Conclusions:**

Diarrhea caused by rotavirus is associated with severe symptoms leading to hospitalization. Consistent with other settings, infants had the greatest burden of disease.

## Introduction

Diarrhea continues to be the second leading cause of death among children under 5 years worldwide and was responsible for approximately 800,000 (~10.5%) of global deaths in this age group in 2015 [1]. Of the 7.6 million global deaths reported among children <5 years in 2010, 9.9% were attributed to diarrheal diseases [2]. This was a remarkable decrease from the 8.9 million reported deaths in 2008 when diarrheal diseases accounted for 15% of deaths [3]. Despite these reductions rotavirus has remained as the most commonest cause of severe gastroenteritis [4] and the estimated decrease in deaths associated with the disease has been reported to range from ~500,000 deaths in 2008 among children <5 years of age, accounting for 5% of total global deaths [5] to ~200,000 in 2015 among the same age group [6], consistent with the WHO Child Health Epidemiology Reference Group (CHERG) estimates [1]. Approximately two-thirds or more of these deaths continue to occur in South Asia and sub-Saharan Africa [1, 2, 4]. In Kenya, rotavirus diarrhea is estimated to cause over 19% (~9,000) of diarrhea hospitalizations, 16% (~1.5 million) of clinic visits for diarrhea and more than 4,000 deaths among children <5 years of age annually [5, 7, 8]. More severe diarrhea and vomiting leading to severe dehydration are common classical symptoms associated with rotavirus disease [9–11].

Currently available rotavirus vaccines have been shown to be effective in reducing the disease burden [12–16]. In July 2014, Kenya introduced rotavirus vaccine as part of the National Immunization Program. Understanding the epidemiology of rotavirus infections in the local setting therefore remains essential for documenting the basis for rotavirus immunization. Furthermore such information is useful in guiding implementation of other concurrent interventions that are effective in the prevention and treatment of diarrhea such as oral rehydration therapy (ORT) inclusive of continued and ideally increased fluid intake and feeding during diarrheal episodes, zinc treatment, and improvements in water and sanitation [17]. In this study, we describe the epidemiology, seasonality, clinical features and factors associated with rotavirus infection among children <5 years of age with non-dysenteric MSD in rural western Kenya prior to rotavirus vaccine introduction.

## Materials and Methods

### Study design

We evaluated data collected from cases enrolled in the Global Enteric Multicenter Study (GEMS), a 4-year; prospective, age-stratified, health center-based matched case-control study of MSD among children aged 0–59 months old residing within a defined and enumerated population [18–20].

### Study setting

**Fig 1:**
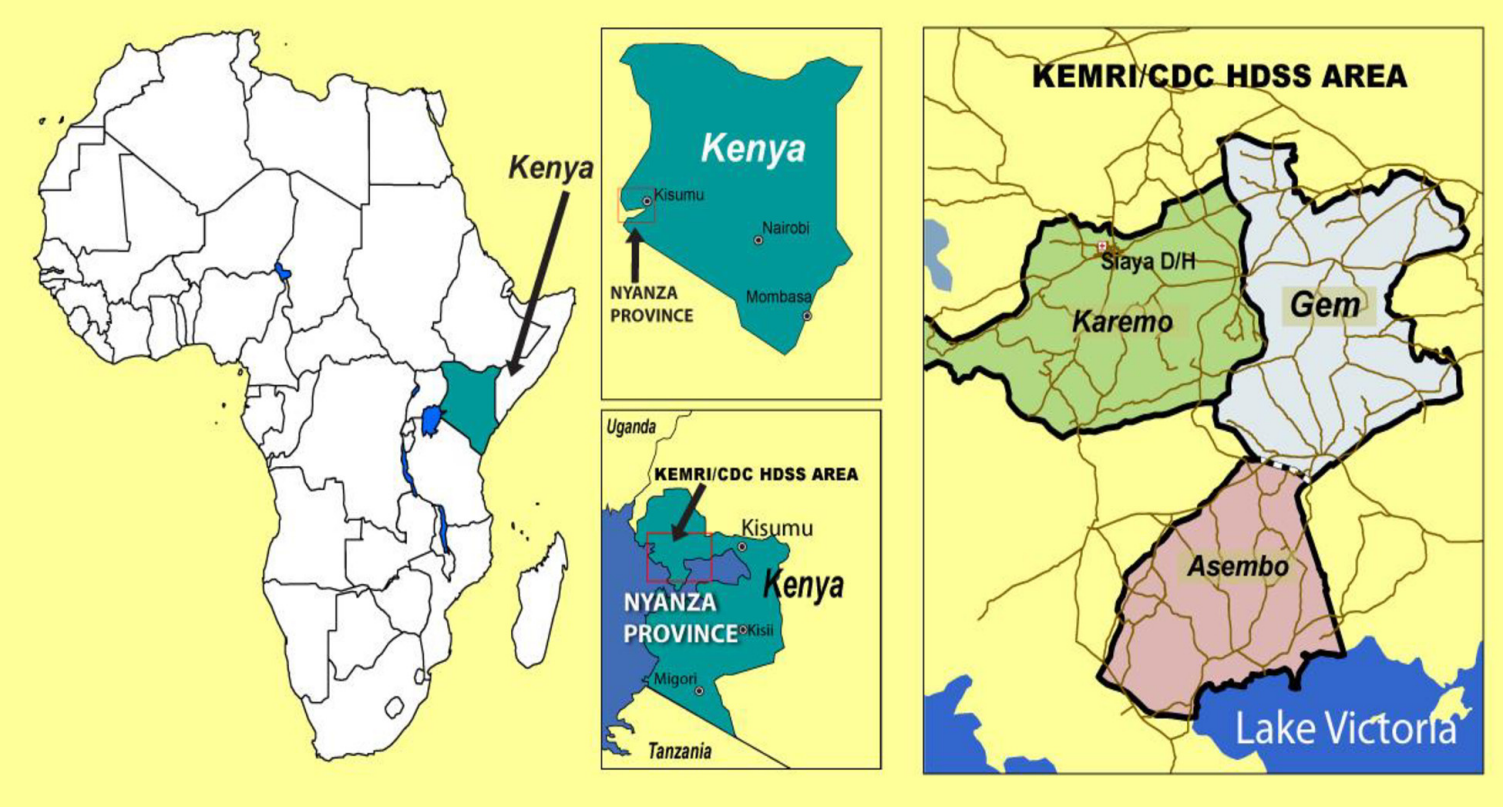
KEMRI/CDC HDSS study area (Asembo, Gem and Karemo) where GEMS Kenya Study was conducted.

The study was conducted in the Asembo, Gem and Karemo communities in Siaya County, (formerly Nyanza province) in rural western Kenya. The Kenya Medical Research Institute (KEMRI) in collaboration with the U.S. Centers for Disease Control and Prevention (CDC) has been operating a health and demographic surveillance system (HDSS) in these communities since 2001, see Fig 1. The study setting has been described further elsewhere [19, 20].

### Study case definition, recruitment and laboratory methods

A non-dysenteric MSD case was defined as a child with ≥3 loose stools in 24 hrs. and one or more of the following: sunken eyes, skin tenting, requiring intravenous rehydration, or hospitalization, who sought care from outpatient or in-patient department of a study sentinel health center (SHC) within 7 days of illness onset. In this analysis, MSD cases with dysentery were excluded.

Caretaker’s maximum education was classified as formal education (completed primary, secondary or post-secondary) or non-formal (incomplete primary, religious education or no education). We classified dehydration as either mild or moderate to severe as follows: a child was considered mildly dehydrated if 2 or more of the following were present: restless or irritable on arrival or at admission to the SHC, sunken eyes, thirsty, drank eagerly; skin pinch goes back slowly (1–2 seconds). A child was considered moderately to severely dehydrated if 2 or more of the following were present: lethargic or unconscious on arrival or at admission to the SHC, sunken eyes, drank poorly or unable to drink, skin pinch goes back very slowly (>2 seconds). Fever was defined by the presence of an axillary body temperature greater than or equal to 38°C or parental perception.

At enrollment, demographic, clinical, epidemiological information and stool samples were collected. Rotavirus VP6 antigen was detected in the whole stool specimen by a well-validated commercial enzyme-linked immunosorbent assay (ELISA) (ProSpecT rotavirus kit, Oxford, Basingstoke, UK). Detailed laboratory methods are described elsewhere [21]. A single home visit ~ 60 days (targeted range 50–90 days) following enrollment was carried out to assess each child’s health outcome from the acute diarrheal illness [8, 18]. Mortality that occurred at any time point between initial enrollment from the SHC and the follow-up visit was recorded.

### Statistical analysis

Data collection and management procedures for GEMS have been described previously [22]. Data were analyzed using SAS version 9.4 (SAS Institute, Inc. Cary, North Carolina, USA).

To compare non-dysenteric MSD cases who tested positive vs. negative for rotaviru**s,** we report proportions and chi square p-values for categorical variables, including mortality recorded at ~60 days follow-up. Medians for continuous variables were compared with Wilcoxon rank sum test. We initially explored the association between each factor and rotavirus positivity among non-dysentery MSD cases one at a time using univariable logistic regression models. Since many of the factors of interest might be highly correlated, we assessed correlation across variables and when present, only the variable with the strongest association with rotavirus positivity was considered for the multivariable model. We then tested for two-way interactions between each of the variables and age because risk factors are likely to be different for infants. We conducted manual backwards stepwise elimination; the final multivariable model included all variables and aimed to include interactions which retained statistical significance at *p*<0.05. We report adjusted odds ratios (aOR) and 95% confidence intervals (CI) from the final model as variables significantly associated with rotavirus. Collinearity was assessed in the final model using condition index as described elsewhere [23, 24].

To assess bias, sensitivity analysis was performed by constructing additional models with various subsets of the data. Children were eligible for re-enrollment as an MSD case after 90 days post enrollment. Therefore, it was possible for some children to be enrolled for more than one episode of MSD. We constructed additional models excluding all observations for the 33 cases who were enrolled more than once, to ensure consistency of results and assess any potential bias. In addition, at enrollment, case stools were tested for a panel of enteric pathogens and thus could have had more than one pathogen identified. We assessed models limited to the 86 cases who had rotavirus as a single pathogen and 159 cases who had rotavirus with other enteric co-infections separately.

Additionally, to further evaluate clinical features’ associated with rotavirus, we applied a 17 point scoring system, referred to in this analysis as “GEMS modified Vesikari score system” which was adapted from the 20 point scale developed by Ruuska and Vesikari [25] to assess severity of rotavirus diarrhea in a separate subgroup analysis which was different with the above models. The score was calculated based on symptoms of diarrheal illness and the child’s characteristics at enrollment. To compare clinical features and modified Vesikari scores we used chi square tests.

Assessment of seasonal patterns was limited to the first three years (36 months) of the study where we had un-interrupted continuous and consistent monthly study enrollments that could support a seasonality analysis. We classified months for this period into cool and rainy (April, May, June, September, October and November) and warm and dry (January, February, March, July, August and December) based on the seasonal patterns in the study area. We compared proportions of rotavirus positivity by season type and computed prevalence odds ratios and confidence intervals using bivariate logistic regression. We further explored seasonality pattern (probability of monthly stool samples testing positive for rotavirus) in a separate logistic regression model for the first three years of data where there was continuity using sine and cosine functions of time [26].

### Verbal cause of death (VA)

VA data collection and analysis methods used in this study has been described elsewhere in detail [19, 27]. In brief, VA interviews were conducted by trained field workers using VA questionnaires. They interviewed the main caregiver of the deceased child on signs and symptoms leading to the death and care seeking behavior during the illness. Information from these questionnaires were processed into Inter- VA -4 (version 4.02) program to obtain most probable/underlying cause of death as further described elsewhere [28].

### Ethics Statement

This evaluation is covered by the GEMS Kenya protocol which was approved by the scientific and ethical review committees of KEMRI (KEMRI protocol # 1155) and the Institution Review Board (IRB) of the University of Maryland, School of Medicine, Baltimore, MD USA (UMB Protocol # H-28327). The IRB for the Centers for Disease Control and Prevention, Atlanta, GA, USA formally deferred its review to the University of Maryland IRB (CDC Protocol # 5038). Written informed consent was obtained in the local dialect (i.e. Dholuo) from all participating caretakers before recruitment of their children into the study. Data were fully anonymized at collection.

## Results

### Enrollment Profile

From January 31, 2008 to September 30, 2012, 1,778 MSD cases were enrolled in GEMS at the Kenya site; 253 were positive for rotavirus. During the first three years of the study when we had consistent enrollments without interruption, 1,476 MSD cases were enrolled. When stratified by in-patient and out-patient type, children <12 months of age compared to children 23 to 59 months of age were at increased risk of rotavirus infection and the risk of infection reduced by increasing child age regardless of patient type. During the three years period, dysentery was significantly less commonly observed among any rotavirus positive (5/217 [2.3%])) compared to negative (106/1,258 [8.4%]) MSD cases, odds ratio = 0.26; 95% confidence interval (CI), 0.10–0.64, p = 0.003. Furthermore the pattern remained similar with dysentery being observed to be less common among rotavirus-positive compared to negative MSD cases when we stratified our analysis by in-patient and out-patient MSD cases. Among both in-patient and out-patient MSD cases, the highest rotavirus isolation occurred in the first year of the study with a decrease among in-patient but a stable rate of isolation among out-patient MSD cases in the second and third years respectively (Table 1).

**Table 1:** Proportion of all rotavirus-positive vs negative stool samples from all GEMS children enrolled with MSD episodes within in-patient and out-patient departments during the first 3 years of non-interrupted enrollment, western Kenya, Jan 2008-Feb 2011.

	In-Patient MSD cases						Out-Patient MSD cases					
Characteristic	Rotavirus positive		Rotavirus negative			P-value	Rotavirus positive		Rotavirus negative			P-value
	(N = 34)		(N = 125)				(N = 183)		(N = 1,134)			
	n	%	n	%	OR95%CI		n	%	n	%	OR95%CI	
Child’s age stratum (in months)												
0–11	23	67.6	54	43.2	†	0.01	114	62.3	482	42.5	5.41 [3.10–9.43]	<0.0001
12–23	10	29.4	37	29.6		0.04	54	29.5	309	27.2	3.99 [2.21–7.23]	<0.0001
24–59	1	2.9	34	27.2		Ref	15	8.2	343	30.2	Ref.	
Median age in months	9 [IQR 6–13]		13 [IQR 8–24]		N/A	0.002	8 [IQR 5–15]		14 [IQR 8–27]			<0.001
Dysentery												
Yes	1	2.9	13	10.5	0.26 [0.03–2.05]	0.201	4	2.2	1041	91.8	0.25 [0.09–0.69]	0.007
No	33	97.1	111	89.5	Ref.		179	97.8	93	8.2	Ref.	
												
Year of study												
Year 1	15	44.1	65	52	Ref.		70	38.3	486	42.9	Ref.	Ref
Year 2	10	29.4	37	29.6	1.17 [0.48–2.87]	0.755	56	30.6	390	34.4	0.99 [0.68–1.45]	0.987
Year 3	9	26.5	23	18.4	1.69 [0.65–4.40]	0.292	57	31.1	258	22.8	1.53 [1.05–2.24]	0.028

† Fisher`s exact test method used

**Fig 2:**
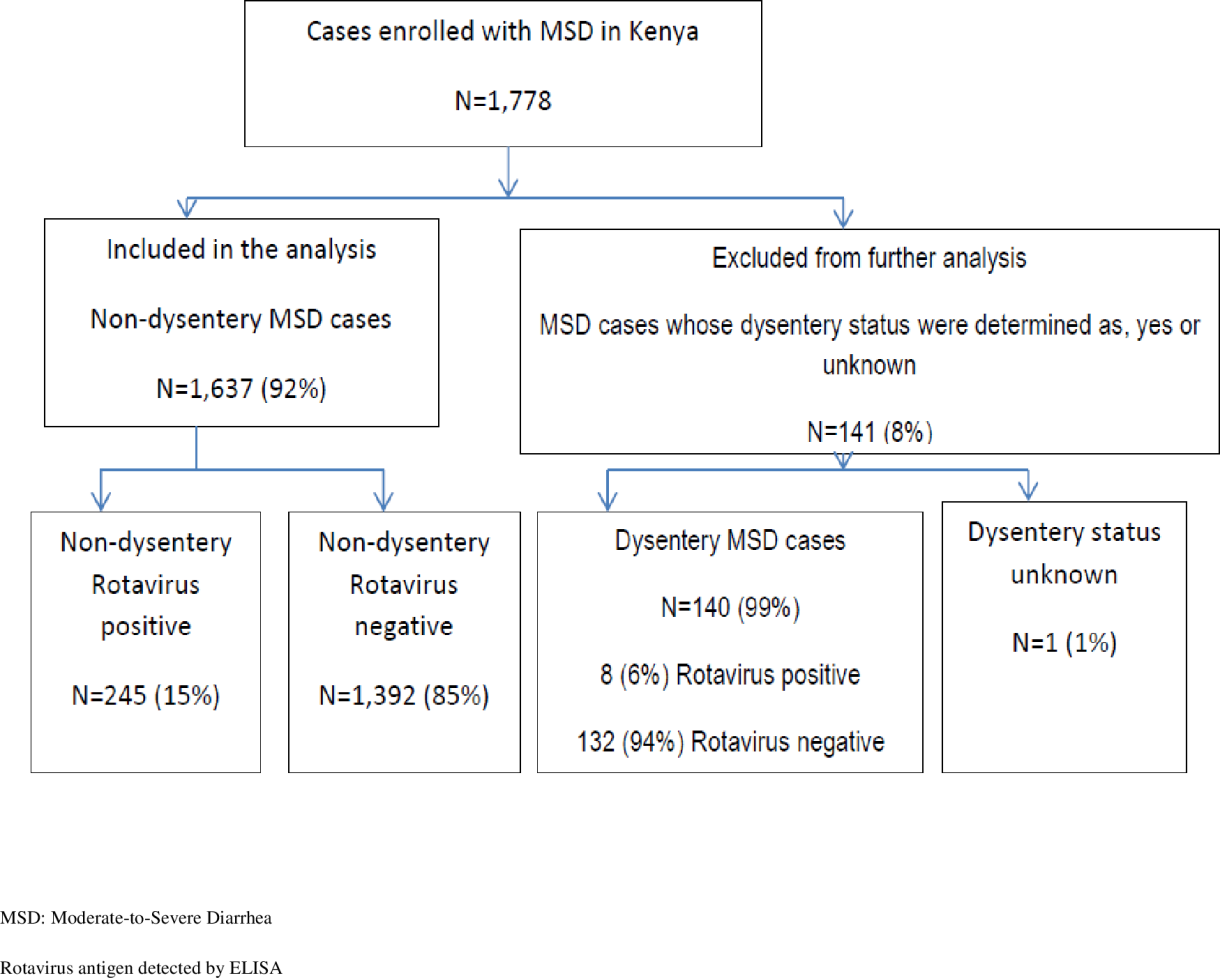
Flow diagram of enrollment of MSD cases in GEMS-Kenya, and the number of non-dysenteric MSD rotavirus-positive and negative cases used for analysis, western Kenya 2008–2012.

All dysentery cases from the four year study period were excluded from further analysis including 8 (3.2%) rotavirus MSD cases, 132 (8.7%) rotavirus negative MSD cases and 1 rotavirus-negative MSD case with unknown dysentery status (Fig 2). A total of 1,637 non-dysenteric MSD cases of whom 245 (15%) were rotavirus-positive and 1,392 rotavirus-negative were included in further analyses as shown in Fig 2.

Breast feeding information presented in the current analysis was only available for the first three years of the study and was available for 1,364 of the 1,476 MSD cases enrolled in that period. In general 441 (32.3%) of the cases had stopped breastfeeding, 867 (63.6%) were partially breastfed and 56 (4.1%) were exclusively breastfed. When we limited our analysis further among 235 cases who at enrollment were aged <6 months and who were ideally expected to be on exclusive breastfeeding, only 50 (21.3%) were exclusively breastfeeding, 180 (76.6%) were partially breastfeeding and 5 (2.1%) had stopped breastfeeding. Exclusive breastfeeding was less frequently reported among caretakers of rotavirus positive (8/59 [13.6%])) compared to negative (42/176 [23.9%]) MSD cases, p = 0.095.

### Factors associated with rotavirus infection

**Fig 3:**
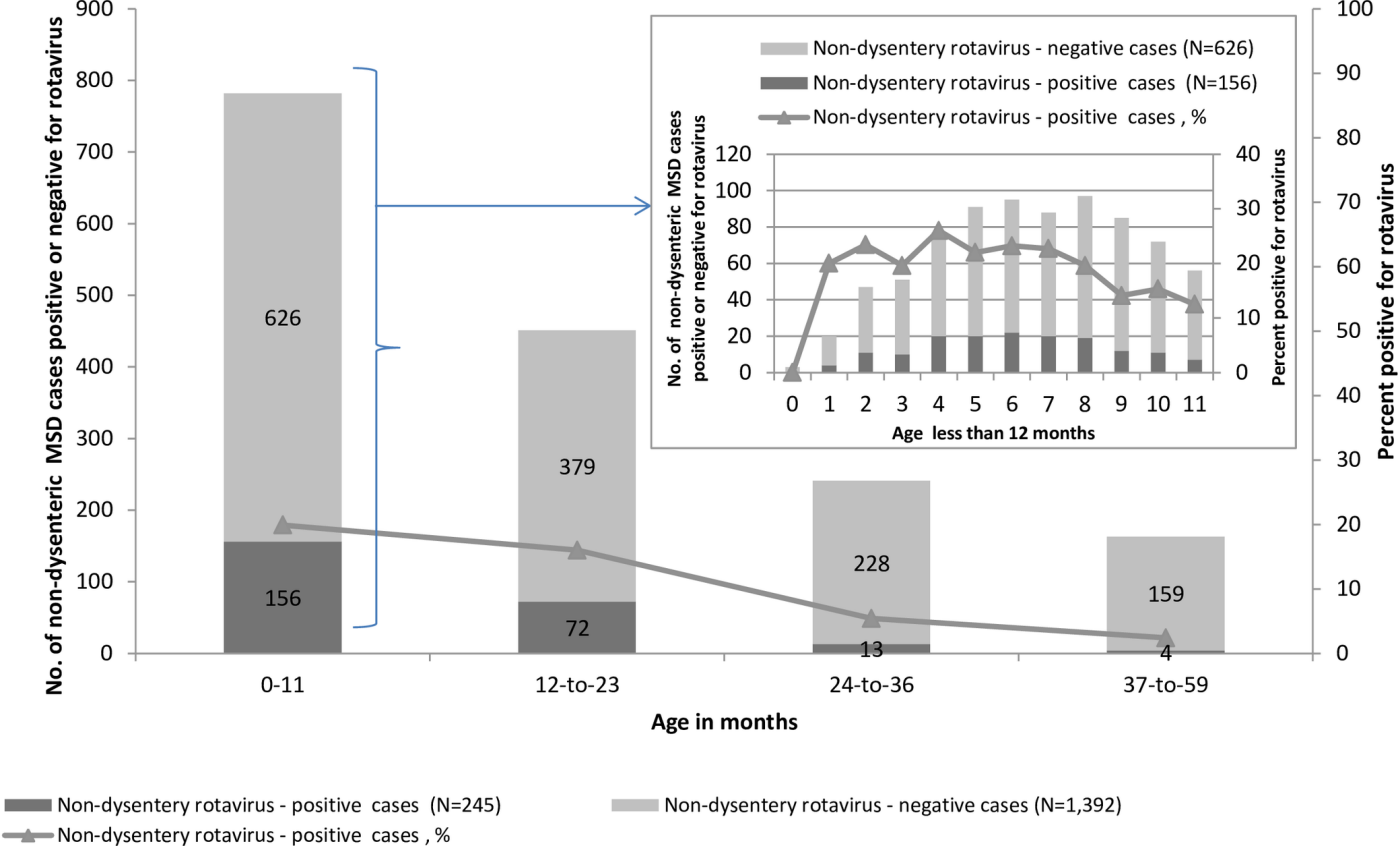
Rotavirus positivity among non-dysenteric MSD cases by age group, western Kenya 2008–2012.

Compared with rotavirus-negative cases, rotavirus-positive cases were significantly younger (median age; 8 vs 13 months *p*<0.0001). The majority (64%) of rotavirus-positive cases were aged 0–11 months as shown in Table 2 and in Fig 3. Further patient demographics, clinical characteristics and laboratory findings are listed in Table 2.

**Table 2:** Bivariate analysis of baseline characteristics of children with rotavirus-positive and rotavirus negative non-dysenteric MSD (n = 1637), western Kenya, 2008–2012.

	Rotavirus-positive		Rotavirus-negative		*P*-value
	N = 245		N = 1,392		
Characteristic	n	%	n	%	
**Child’s age stratum (in months)**	** **	** **	** **	** **	** **
0–11	156	63.7	626	45.0	<0.001
12–23	72	29.4	379	27.2	<0.001
24–59	17	6.9	387	27.8	
Median age in months	8 [IQR 5–14]		13 [IQR 7–25]		<0.001*
**Gender**					
Female	120	49.0	591	42.5	0.058
**Caretakers Education**^**∫∫**^					
Formal education	125	51.0	606	43.6	0.118
Non-formal education	120	49.0	784	56.4	
**Clinical symptoms in the child at enrollment (% yes)**					
**Vomiting ≥3 times/24hrs**	171	69.8	627	45.0	<0.001
**Maximum no. of loose stools/24hrs**					
≥ 7/24hrs	78	31.8	333	23.9	0.009
≤ 6/24hrs	167	68.2	1059	76.1	
**Unable to drink**	16	6.5	44	3.2	0.011
**Sunken eyes**	238	97.1	1307	93.9	0.047
**Irritable/restlessness**	179	73.1	856	61.5	<0.001
**Lethargy**	35	14.3	127	9.1	0.013
**Child’s mental status abnormal**^§§^	186	75.9	874	62.8	<0.001
**History of Fever (as observed by caretaker)**	173	70.6	1076	77.3	0.024
**Dehydration**^**§**^					
Moderate-to-severe^‡ ‡^	55/241	22.8	250/1333	18.8	0.14
Mild ^‡^	186/241	77.2	1083/1333	81.2	
**Child was admitted to hospital**	37	15.1	134	9.6	0.01
**IV fluid given**	43	17.6	155	11.1	<0.005

*p value based on Wilcoxon Rank Sums test

∫∫ = denominator = 1,635;

^**§§**^ = A child’s mental status was considered abnormal if restlessness or irritable or lethargy or unconsciousness was present or observed by clinician at enrollment

^**§**^ A child was considered dehydrated if either mild or moderate to severe dehydration symptoms were present as described by the following classifications

^‡ ‡^ A child was considered moderately to severely dehydrated if 2 or more of the following were present: lethargic or unconscious on arrival/admission; sunken eyes; drank poorly or unable to drink; skin pinch—goes back very slowly (>2 seconds)

^‡^ A child was considered mildly dehydrated if 2 or more of the following were present: Restless/irritable on arrival/admission; sunken eyes; thirsty, drank eagerly; skin pinch—goes back slowly (1–2 seconds); ¶ = denominator is 1,574

From birth prevalence increased to 20% at age 1 month and remained at more or less at the same level through age 8 months, with a sharp drop-off in month 9, 10 and 11, see Fig 3. Although some of the SHC did not have full in-patient facilities, patients in this study who required more time for rehydration at the facility were retained in an improvised in-patient unit sometimes called “ORS corner” for a few hours while undergoing further observation while being rehydrated either through IV fluid or ORS. However in situations when further complications or such patients required full hospitalization then they were referred appropriately to the next level or superior health centers or hospitals with full in-patient services. Hospitalization was more commonly observed among MSD patients <1 year of age who were rotavirus-positive versus negative (67.6% [25/37] vs. 46.3% [62/134], *p*<0.035) and among all rotavirus-positive vs negative MSD cases regardless of age (15.1% [37/245] vs. 9.6% [134/1,392], *p* <0.013). However, the overall length of stay in hospital was generally similar (median = 2 days) for hospitalized rotavirus-positive and negative MSD cases. Other factors that were associated with rotavirus MSD in univariable analysis include; vomiting ≥3 times in 24 hours, ≥7 loose stools in 24 hours, unable to drink, sunken eyes, irritability/restlessness, lethargy, abnormal mental status and IV fluids given (Table 2).

### Univariable and multivariable analysis

In univariable analysis, compared with rotavirus-negative cases, rotavirus-positive cases were significantly more likely to present with sunken eyes (238/245 (97.1%) vs. 1,307/1,392 (93.9%), OR = 2.21, 95%Confidence interval (CI): 1.01–4.84, p = 0.047); to be restless (179/245 (73.1%) vs. 856/1,392 (61.5%), OR = 1.7,95%CI: 1.25–3.0, p = 0.0006); to have abnormal mental status (186/245 (75.9%) vs. 874/1,392 (62.8%), OR = 1.87,95%CI:1.37–2.55, p<0.0001) and to be unable to drink (16/245 (6.5%) vs. 44/1,392 (3.2%), OR = 2.14,95%CI:1.19–3.86, p = 0.011) and to be hospitalized upon seeking care (37/245 (15.1%) vs. 134/1,392 (9.6%), OR = 1.67, 95%CI:1.13–2.47,p = 0.01. Other factors associated with rotavirus infection included age, vomiting, number of loose stools, hospitalization and administration of IV fluids upon seeking care (Table 2). Number of loose stools during the diarrheal illness were significantly correlated with age, while intravenous fluid was correlated with fever, dehydration and admission. Receipt of IV fluids and hospitalization were highly correlated and could not be included in the model together. Only receipt of IV fluids was retained for model selection. In multivariable analysis, younger age and vomiting ≥3 times per day remained significantly associated with rotavirus infection (Table 3). We found no statistically significant interactions between any of the variables that qualified for the multivariable model. In addition, sensitivity analysis of models excluding repeat enrollments and subsets of single and multiple pathogen positivity yielded the same conclusions presented for the overall analysis, thus, data are not shown.

**Table 3:** Crude and adjusted odds ratios of factors associated with rotavirus among children <5 years of age with non-dysenteric MSD, western Kenya, 2008–2012.

Characteristic†	Odds ratio (OR) for being ELISA positive for rotavirus diarrhea [95% confidence interval (CI)]	
Child’s age stratum (in months)	Unadjusted Odds Ratio (OR) 95% CI	Adjusted Odds Ratio (aOR) 95% CI
0–11	5.67 (3.38–9.51)	5.29 (3.14–8.89)
12–23	4.32 (2.50–7.47)	4.08 (2.35–7.07)
24–59	Ref ††	Ref
**Gender**		
Female	1.3 (0.99–1.71)	
Male	Ref	Ref
**Clinical symptoms in the child at enrollment**		
**Vomiting ≥3 times/24hrs**		
Yes	2.82 (2.10–3.78)	2.66 (1.98–3.57)
No	Ref	Ref
**Maximum no. of loose stools/24hrs**		
≥ 7/24hrs	1.49 (1.10–2.00)	
≤ 6/24hrs	Ref	Ref
**Dehydration**		
Moderate-to-severe	1.28 (0.92–1.78)	
Mild	Ref	Ref
**IV fluid given**		
Yes	1.7 (1.17–2.46)	
No	Ref	Ref

† 6 variables initially entered into the model out of which 2 (above) were associated with rotavirus positivity. Variables dropped were 4 namely; Child offered IV fluid, dehydration, Maximum stool in 24hrs and gender

^††^Ref denotes the referent group

Finally using the GEMS modified Vesikari score at enrollment, we found that rotavirus-positive compared to negative cases were more likely to have a higher median Vesikari score (9 vs. 8), *P*<0.01 and were commonly observed to present with more severe disease symptoms. (Table 4).

**Table 4:** Numerical (GEMS modified) scoring system for severity of diarrhea among rotavirus-positive and negative children with non-dysenteric MSD, western Kenya, January 31, 2008-September 30, 2012.

Child characteristics	Points assigned	Rotavirus-positive		Rotavirus-negative		p-value
	(N = 17)	N = 245		N = 1,392		
		N	%	n	%	
**Duration of diarrhea (days)**						
1–4	1	221	90.2	1,247	89.6	0.954
5	2	14	5.7	85	6.1	
≥6	3	10	4.1	60	4.3	
**Max no. diarrhea/24 hrs.**						
3–6	2	167	68.2	1,059	76.1	0.010
≥7	3	78	31.8	333	23.9	
**Vomited 3+ times/24 hrs.**						
Yes	3	171	69.8	627	45.0	<0.001
No	0	74	30.2	765	55.0	
**Fever** †						
<37.0	0	82	33.5	550	39.5	<0.001
37.1–38.4	1	128	52.2	534	38.4	
38.5–38.9	2	17	6.9	112	8.1	
> = 39	3	18	7.4	195	14.0	
**Dehydration**††						
Moderate/Severe‡‡	3	55	22.8	250	18.8	0.156
Mild‡	2	186	77.2	1,083	81.2	
**Treatment**						
Out-patient without IV fluid	0	198	80.8	1,215	87.3	0.023
Out-patient with IV fluid	1	10	4.1	43	3.1	
Hospitalization with or without IV fluid	2	37	15.1	134	9.6	0.013
**Median Score** †††	N/A	9 [IQR] 8–10		8 [IQR] 6–10		<0.001

^†^ Denominator = 1,636

^††^ Denominator = 1,574

^†††^ Median calculated for children not missing information (rotavirus-positive = 241 and rotavirus negative = 1,332)

^‡^ A child was considered mildly dehydrated if 2 or more of the following were present: Restless/irritable on arrival/admission; sunken eyes; thirsty, drank eagerly; skin pinch—goes back slowly (1–2 seconds)

^‡‡^ A child was considered moderately to severely dehydrated if 2 or more of the following were present: lethargic or unconscious on arrival/admission; sunken eyes; drank poorly or unable to drink; skin pinch—goes back very slowly (>2 seconds)

### Mortality at sixty-day follow-up

Overall 1,580 (96.5%) of the 1,637 enrolled cases had their caretakers successfully interviewed at 60-day follow-up. A higher proportion of rotavirus-positive (11/242 [4.5%]) compared to negative (51/1,476 [3.5%]) cases died before the home visit follow-up interview, although the differential in case fatality was not statistically significant (*P* = 0.36). VA cause of death information was available for 60 MSD cases that including rotavirus-positive (10 [17%]) and negative (50 [83%]) cases that had died before follow-up. The causes of death among the 10 rotavirus-positive cases included: diarrhea 5 (50%), HIV 2 (20%), malaria 1 (10%), TB 1 (10%) and parasitic disease 1 (10%). The causes of death among the 50 rotavirus-negative cases included; diarrhea 13 (26%), HIV 13 (26%), malaria 13 (26%), malnutrition 5 (10%), pneumonia 3(6%), TB 2 (4%) and measles 1 (2%). The median time from discharge to death was 12 (IQR) 7–18 days for rotavirus-positive MSD cases and 14 (IQR) 7–33 days for rotavirus-negative MSD cases P = 0.56

### Seasonality of rotavirus

**Fig 4:**
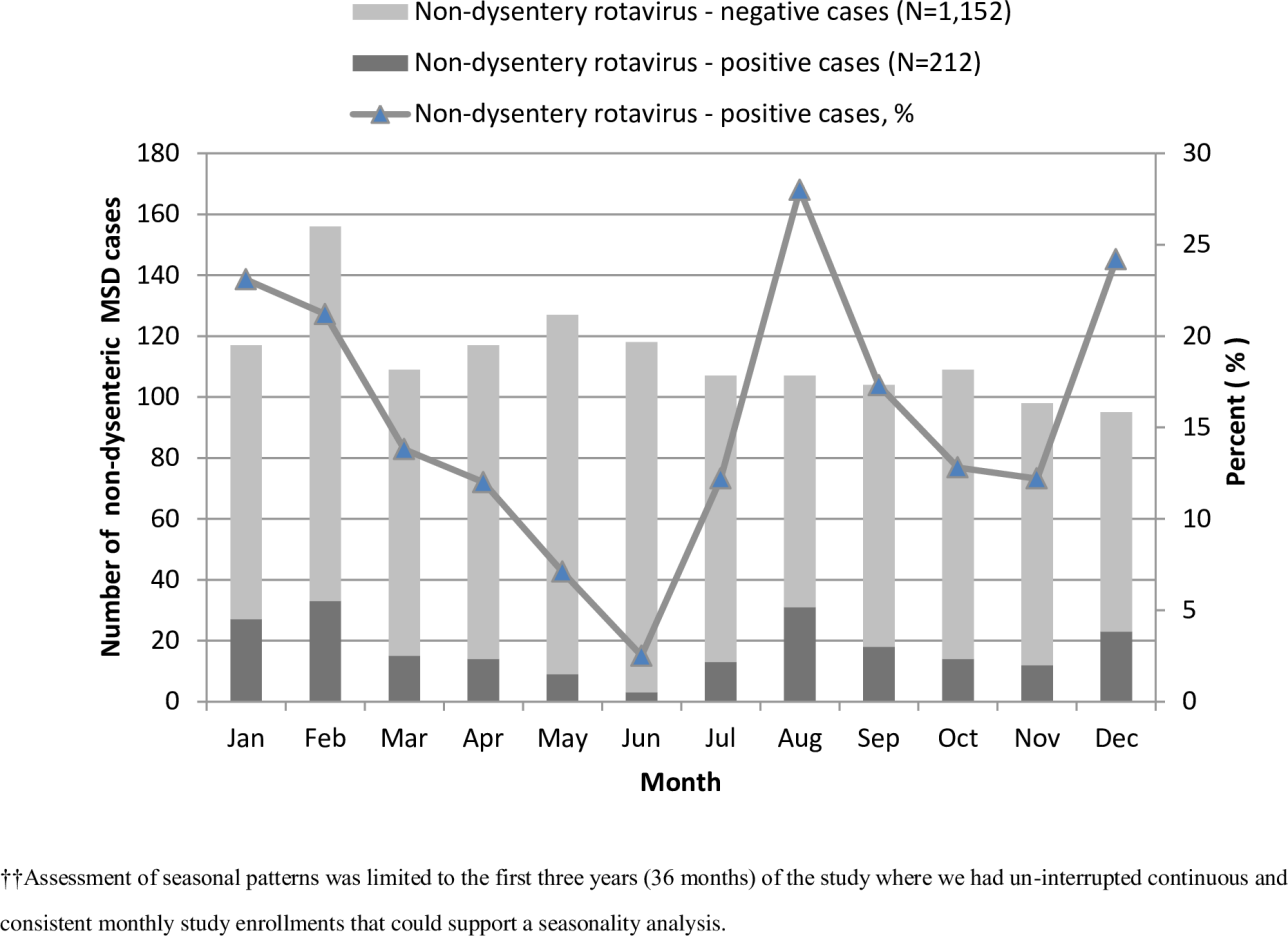
The seasonal distribution of rotavirus-positive compared with negative cases among children with non-dysenteric moderate-to-severe diarrhea (MSD) in western Kenya, January, 2008- February, 2011.

For the first 3 years of the study, prevalence of non-dysenteric rotavirus-positive MSD cases by year of study ranged from 14.2% (85/598) in the first year to 13.8% (63/457) in the second year and finally to 20.7% (64/309) in the third year. Stool samples collected during usually warm and dry months compared with usually cool and rainy months were twice as likely to be rotavirus-positive, (142/691 [20.5%] vs 70/673 [10.4%]; OR = 2.16, 95%CI, 1.58–2.96). Although we found that the proportion of cases positive for rotavirus was highest in August, December, January and February as shown in Fig 4, when we tested seasonality in a model using sine and cosine functions of time, it revealed that the pattern of seasonality (probability that a stool sample would test positive for rotavirus) was significantly inconsistent across the 3 years of the study period (*P*-value = <0.0001).

## Discussion

In this study, we describe the epidemiology and factors associated with rotavirus infection among patients <5 years of age in rural western Kenya, using comprehensive baseline and 60-day follow-up data. Our study finding that the risk of rotavirus infection was highest among infants—an age group that accounted for more than 60% of rotavirus infections in our study is consistent with other observations already made from other sub-Saharan African and Asian countries [8, 29]. However, we did not detect any rotavirus cases in neonates but few neonates with MSD were enrolled in our study.

Clinical assessment, treatment and decision for admission of diarrhea patients to a hospital can be influenced by differences in socio-economic factors, or by clinician’s attitudes. In the current study, rotavirus cases among children <1 year also had an increased likelihood of hospitalization compared to rotavirus-negative infants, an observation we argue may have not been biased by test results, since stool testing is batched and results are not available to clinicians who decide on hospitalization and treatment until later in the clinical course. Our current study demonstrates that the proportion of infants infected with rotavirus among out-patients was higher compared to children 24–59 months of age. Furthermore among in-patient study population, infants were at the greatest risk of hospitalization, followed by children 12–23 months of age compared to children 24–59 months of age. Consistent with other studies, rotavirus diarrhea has been shown to be a major cause of hospitalization mostly in infants [30].

In our current study as seen elsewhere [11], clinically diagnosed fever by our study clinicians was associated with rotavirus diarrhea. However, when we asked caretakers separately whether they had observed history or presence of fever during their child’s diarrheal episode, the caretakers reported fever was seen to be more common among rotavirus-negative compared to positive MSD cases suggesting that caretakers may have not been either keenly observant of fever or may have not recognized fever to be related to the diarrheal illness. Our previous healthcare utilization and attitude survey, conducted before hospital-based surveillance began, revealed that fever was not among the factors that prompted caretakers to seek care for their child`s diarrheal illness[20]. This observation may in part explain the lack of appreciation by caretakers in this community that fever and diarrhea may be related.

Our modified GEMS clinical scoring system adapted from the Vesikari score system [25] enabled us to assess the severity of rotavirus compared to non-rotavirus MSD. As shown in our study and consistent with findings across many settings, in young children, rotavirus disease is characterized by diarrhea, vomiting, and severe dehydration [31]. The overall median severity score was significantly higher among rotavirus positive than negative cases and similar observations have been made in other studies elsewhere [32, 33]. We also observed that diarrhea severe enough to lead to hospitalization was prevalent among the infants and young children diagnosed with rotavirus disease—a finding that is consistent with observations previously made elsewhere[33] and also by the World Health Organization [31]. Overall our current study shows that there was generally very little exclusive breastfeeding, with almost 80% of MSD cases aged <6 months either partially breastfeed or had stopped breastfeeding. Furthermore, although not statistically significant, exclusive breastfeeding was less frequently reported among caretakers of rotavirus positive (13.6%) compared to negative cases (23.9%)—an observation which is not dissimilar to that made in other settings[33]. These findings may suggest that maternal antibodies could be insufficient to protect against severe rotavirus illness. Second, the age-associated differences observed in rotavirus prevalence, including the less common rotavirus occurrence among breastfed children aged <6 months and those aged <4 months as observed in our study is worth noting. Infants receive passive protection from trans-placental and breastmilk antibodies for the first 6 months of life. Infants who continue to exclusively breastfeed are less likely to be exposed to pathogens than infants on mixed feedings during the six months of age [34]. The child’s first exposure to a rotavirus infection during the first six months of life often results in severe disease due to lack of antibodies to fight the infection and is more likely to lead to hospitalization as our current findings suggest since passive immunity wanes and natural immunity acquired from such exposure builds up; subsequent infections tend to produce milder illness which may be characterized by less severe disease and admission to hospital as suggested by our data. Therefore as we have observed in this study and as shown from other settings, the rate of illness declines as the child’s age increases and probably as children acquire immunity increasingly from subsequent rotavirus infections[35].

Active, sentinel surveillance of rotavirus diarrhea could provide useful data that can guide the interpretation of diarrheal disease trends following rotavirus vaccine introduction in settings such as Kenya. During the first 3 years of year surveillance, we found rotavirus prevalence rate of 14.2% in year 1 and which remained stable in year two (13.8%) but increased to 20.7% in year 3 among children seeking care at a hospital or health center for non-dysenteric MSD. Our prevalence rate of rotavirus among MSD patients <5 years in year one and two of the surveillance is slightly higher but remains comparable to a prevalence of 12% reported from a population-based surveillance study conducted in both urban slums in Nairobi and rural western Kenya [36] and to 13.5% from a similar study conducted in Nigeria [37]. Furthermore, our observed prevalence especially in year 3 is similar to that observed in other hospital-based studies conducted in the neighboring countries like Tanzania (21%) [10]and Ethiopia (21%) [38]. However, our reported prevalence of rotavirus is relatively lower than other observations from other studies conducted in Africa; for example 33% in Burkina Faso[9] and 45% in Uganda[32]. The differences observed across these studies may be explained by differences in study methodologies including variations in seasonality, study periods, study populations and possibly laboratory techniques. However the observed variations in rotavirus rates further highlights the need and importance of national and regional standardization of rotavirus surveillance using different approaches and techniques that can support comparison and monitoring of rotavirus trends post-vaccine implementation and to perform vaccine impact assessment and cost-effective analyses.

Studies from other countries within and outside Africa have shown that rotavirus vaccines are safe, effective and cost-effective interventions against severe rotavirus disease [29]. Following implementation of rotavirus vaccination, remarkable declines in overall diarrhea occurrence and hospitalization associated with rotavirus diarrhea have occurred in a number of both developed and a few developing countries [39–41]. Vaccination is currently the best way to prevent severe rotavirus illness, particularly in settings such as our study area where access to medical care is limited or sometimes unavailable [31] and ORT use is low[20, 39]. Furthermore in countries with poor immunization programs, as in many African countries, administration of vaccine doses may be delayed. The cost-effectiveness of the rotavirus vaccine declines rapidly with a delayed administration of the first rotavirus vaccine dose which highlights the need for strong immunization programs. With support from GAVI, in July 2014, Kenya implemented routine rotavirus vaccination under the country’s national infant immunization program. To realize its full life-saving potential, rotavirus vaccination must reach all vaccine-eligible children.

Although it has been estimated that rotavirus vaccine introduction in Kenya could prevent more than 5,000 hospitalizations and over 800,000 clinic visits among children <5 years annually [42], results would be optimized when complementary interventions such as increasing use of oral rehydration salts (ORS) and exclusive breastfeeding among children < 6 months and those with diarrhea are also strengthened. Dehydration can be reversed through oral rehydration therapy (ORT) (that is continued feeding and fluids including breastfeeding and ORS use at home) or, if more serious, through hospitalization and IV fluids. Furthermore ORT is important in management of rotavirus diarrhea as antibiotics or other drugs have no known benefit on treating such an illness or acute watery diarrhea due to cholera, cryptosporidiosis, and many other similar illnesses. Our finding that there was no difference between all-cause deaths among children with rotavirus compared to non-rotavirus MSD at sixty day follow-up is an important observation that could possibly highlight that mortality is not associated with rotavirus in hospital based studies. This is possible because detection of rotavirus generally requires a visit to clinic, which presumably would lead to rehydration, but non-detected rotavirus cases that did not make it to a clinic would be less likely to access rehydration and may die in the community without seeking care. When examining the verbal autopsy data, we were not powered to compare diarrhea deaths between rotavirus and non-rotavirus MSD cases at the 60-day follow-up visits. However our VA data presented in this analysis has shown that deaths from rotavirus-positive compared to those from negative MSD cases occurred a few days after enrollment which further suggests that children who reach a healthcare facility with acute rotavirus diarrhea may recover sooner than non-rotavirus MSD cases. Our findings further suggests that diarrhea was the leading cause of death among children with moderate-to-severe diarrhea in our study population—an observation that may suggest that diarrhea continues to be a leading cause of morbidity and mortality in this setting.

Our study is subject to biases and limitations. Data from this study may not be generalized to all children <5 years in Kenya as it was conducted in a single rural site in western Kenya. Also, our modified Vesikari score needs to be interpreted with caution. In our study, information on the duration of vomiting and the maximum number of episodes of vomiting over a 24-hour period was not collected because the caretakers were interviewed only at study enrollment. The incomplete capture of information on vomiting in our study did not allow us to calculate the full 20-point Vesikari score. We resolved this by modifying the Vesikari score based on GEMS data and we were able to construct a 17-point scoring system instead.

As rotavirus vaccine is introduced into the Kenya national immunization program, monitoring its impact on diarrheal disease burden, clinical presentation, and seasonality will be important.
